# Blend Hybrid Solid Electrolytes Based on LiTFSI Doped Silica-Polyethylene Oxide for Lithium-Ion Batteries

**DOI:** 10.3390/membranes9090109

**Published:** 2019-08-27

**Authors:** Jadra Mosa, Jonh Fredy Vélez, Mario Aparicio

**Affiliations:** 1 Instituto de Cerámica y Vidrio (CSIC), C/Kelsen, 5, 28049 Madrid, Spain; 2 National Institute of Chemistry, Hajdrihova 19, 1000 Ljubljana, Slovenia

**Keywords:** hybrid organic-inorganic, solid electrolytes, Li-ion batteries, sol-gel, coatings

## Abstract

Organic/inorganic hybrid membranes that are based on GTT (GPTMS-TMES-TPTE) system while using 3-Glycidoxypropyl-trimethoxysilane (GPTMS), Trimethyletoxisilane (TMES), and Trimethylolpropane triglycidyl ether (TPTE) as precursors have been obtained while using a combination of organic polymerization and sol-gel synthesis to be used as electrolytes in Li-ion batteries. Self-supported materials and thin-films solid hybrid electrolytes that were doped with Lithium bis(trifluoromethanesulfonyl)imide (LiTFSI) were prepared. The hybrid network is based on highly cross-linked structures with high ionic conductivity. The dependency of the crosslinked hybrid structure and polymerization grade on ionic conductivity is studied. Ionic conductivity depends on triepoxy precursor (TPTE) and the accessibility of Li ions in the organic network, reaching a maximum ionic conductivity of 1.3 × 10^−4^ and 1.4 × 10^−3^ S cm^−1^ at room temperature and 60 °C, respectively. A wide electrochemical stability window in the range of 1.5–5 V facilitates its use as solid electrolytes in next-generation of Li-ion batteries.

## 1. Introduction

Lithium-ion batteries have growth potentially since their commercialization by Sony in 1990. Lithium-ion batteries (LIBs) present several advantages, such as high energy density, self-discharge, high life cycling, etc., when compared with other energy storage devices [1,2,3,4,5,6]. Lithium-ion batteries are important for a wide variety of applications, such as consumer electronics, electric/hybrid electric vehicles, stationary energy storage systems, etc. The performance of lithium-ion batteries depends on the materials that are used. One materials challenge is the development of safer and more reliable electrolytes to replace that currently used in order to perform long lifespan and high performance LIBs [7,8,9,10,11,12,13]. The electrolyte requirements must combine suitable ionic conduction, adequate thermal and chemical stability and compatibility with electrode materials [8]. Commercial batteries are based on lithium salts that contain organic solvents; these liquid electrolytes that need the use of separators must to be optimized in order to obtain high performance and high ionic conductivity. The use of a solid electrolyte eliminates the need for containment of the liquid electrolyte and separators, which simplifies the cell design, as well as improves safety and durability [14]. All-solid-state batteries are new type of Li-ion battery, and in last year’s had been demonstrated their adequate response in terms of safety, high stability energy-storing devices with higher energy densities. However, the use of such batteries is limited due to their lower ionic conductivity at room temperature of the solid electrolyte and their high impedance at the electrode/solid electrolyte interface, which hinders fast charging and discharging [7,8,9,10,11,12,13,15]. Several strategies have been described in order to enhance the ionic conductivity of solid electrolytes, while using glassy materials, such as NASICON-type, garnet-type, perovskite-type, LISICON-type, LiPON-type, Li_3_N-type, sulfide-type, argyrodite-type, anti-perovskite-type, and many more when compared with other energy storage devices, l [8,9,10,11,12,13,14,15]. Several reviews have addressed and classified solid electrolytes according their chemical nature, glassy or polymer, ionic conductivity, and cycleability [9,14,15,16,17,18]. Concerning polymer solid electrolytes, different polymer precursor have been usually used as poly(ethylene)oxide (PEO), poly-(propylene)oxide (PPO), polyacrylonitrile (PAN), poly(methyl methacrylate) (PMMA), poly(vinylidenefluoride) (PVDF) doping with different lithium salts, such as lithium perclorate (LiClO_4_), lithium hexafluorophosphate (LiPF_6_), lithium tetrafluoroborate (LiBF_4_), and lithium triflate (LiSO_3_CF_3_) [7,8,9,10,11,12,13,14]. In view of the good solvation property of PEO with lithium salts, PEO-based solid electrolytes are the most widely investigated system. Still, not much improvement in the ionic conductivity values for PEO-based solid electrolytes has yet been achieved. Another important modification is the addition of ceramic fillers, such as titania, alumina, silica, zirconia, etc., to improve the ionic conductivity and mechanical stability [7,19].

Another inconvenience in the used of PEO based solid electrolytes is that, presently, some crystallinity affects the mobility of Li ions and decreases ionic conductivity at room temperature. Hybrid organic/inorganic solid materials have been used as solid electrolytes, because they present high ionic conductivity at low temperatures, along with good thermal and mechanical properties [20,21,22,23,24,25,26,27]. The incorporation of inorganic domains within the polymer PEO chains avoiding the crystallization of PEO chains increases ionic conductivity at room temperature and facilitates lithium ion mobility.

In a previous work [28], nanostructured silica-poly(ethylene)oxide-lithium bis(trifluoromethanesulfonyl)imide (silica-PEO-LiTFSI) hybrid organic-inorganic solid electrolytes were prepared via organic polymerization and sol-gel chemistry. The synthesis strategy that was chosen was based on the preparation of a hybrid material class II with covalent bond between organic and inorganic components. The results showed that the ionic conductivity increases with di-epoxy precursor (EDGE) content up to 2.6 × 10^−5^ S/cm at room temperature with enhanced mechanical and thermal stability. According these interesting findings, this hybrid solid electrolyte preparation route is newly investigated while using a tri-epoxy organic component (Trimethylolpropane triglycidyl ether (TPTE)) instead of EDGE in order to improve the structure-conductivity relationship. It is expected that presence of additional ether groups in the organic component facilitates the ionic conduction mechanisms, which enhances ionic conductivity. However, finding the balance between organic crosslinking degrees and ionic mobility is necessary. On one hand, a greater proportion of polyether chains can generate highly cross-linked structures with high ionic conductivity that inhibits the crystallization of PEO chains. On the other hand, longer polymerization degrees can impede the processes of ionic mobility and, therefore, decreases the conductivity. In this case, [Li]/[O] ratio up to 0.10 were implemented, because, as it was previously reported [28], a higher [Li]/[O] ratio led to a decrease in the number of free Li ions, which increases the degree of cross-linking and the formation of Li^+^-TFSI^-^ ion-pairs.

## 2. Materials and Methods

### 2.1. Materials

3-Glycidoxypropyl-trimethoxysilane (GPTMS) (98%), EGDE (99%), and trimethyltriethoxysilane (TMES) (95%) were used as received and purchased from ABCR Company. n-Methylimidazole (n-MI,99%) and LiTFSI (99.95%) from Sigma-Aldrich was used as an initiator for the epoxy group copolymerization and lithium salt, respectively. LiFFSI was dried at 120 °C in a glovebox. Absolute ethanol (99.5%) from Panreac was distilled (Karl–Fischer titration 55 ppm H_2_O) and was used as solvent.

**Figure 1:**
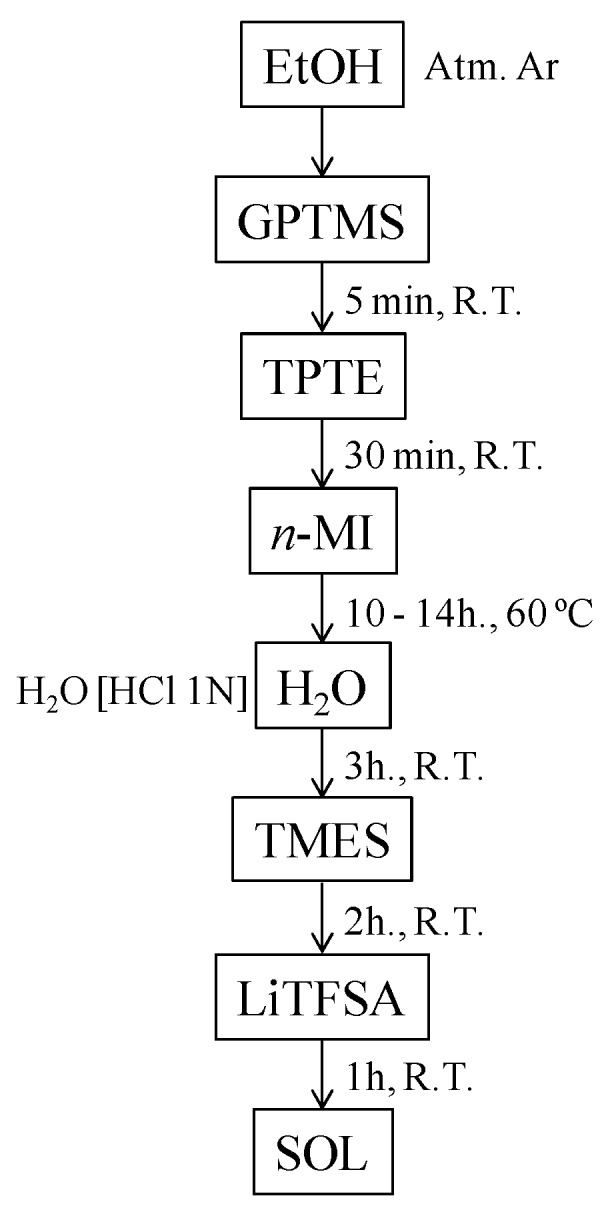
Schematic flux diagram for the synthesis of sols 3-Glycidoxypropyl-trimethoxysilane- Trimethyletoxisilane-Trimethylolpropane triglycidyl ether (GTT).

The synthesis procedure consists in the initial formation of the organic network and, then, the sol-gel reactions (hydrolysis and condensation) forming inorganic networks are promoted, according to the procedure that was followed for the development of the termed “star-branched silica based architectures” [25,28,29]. The preparation of the materials consists of three stages: (i) formation of the organic network; (ii) formation of inorganic environments (sol-gel reactions); (iii) blocking of hydrolyzed groups, uncondensed Si-OH groups, with trimethyltriethoxysilane (TMES), in order to avoid the effect of proton conduction on the conductivity measurement; and, (iv) Once the hybrid structure is formed, the lithium salt (LiTFSI) is added to obtain the desired Li-ion conductive material. The flux diagram describing synthesis strategy for the preparation of GTT: GPTMS/TMES/TPTE compositions are shown in the Figure 1.

Five compositions of the GTT system (Table 1) have been synthesized, varying the ratio between the precursors (GPTMS and TPTE). [Li]/[O] ratio was fixed to 0.10 (based on a previous work [28]), and also non-doped Li composition was studied for comparison.

The materials have been processed as coatings while using the immersion-extraction process and as self-supported membranes. The coatings were deposited on soda-lime glass slides (2.5 × 7 cm^2^) and they were processed at room temperature inside a glove box (Ar) that was equipped with a dip-coater. Extraction speeds between 4.5 and 20 cm min^−1^ were used. The coatings were dried at room temperature for 30 minutes and, subsequently, they were thermally treated at 100 °C for 12 h in an oven (HOBERSAL Model JB-15) (with a constant heating ramp of 1 °C/min.) inside the glove box to complete the drying and sintering of the material. The preparation of the self-supported membranes was based on the casting of the sol in Teflon molds inside the glove box, allowing for the solvent to evaporate for several days at room temperature and, subsequently, treated at 60 °C for 24 h. The membranes were demoulded and treated thermally at 100 °C in order to accelerates condensation of inorganic precursor and consolidate the hybrid structure while using a constant heating ramp at 1 °C/min. for 12 h in an inert atmosphere (Ar).

### 2.2. Methods

The viscosity of sols was measured by a Vibro Viscometer AX-SV-34 (A&D Ltd. Co., Tokyo, Japan) at room temperature. Coating thickness was obtained by spectral Ellipsometric measurements while using a Variable Angle Spectroscopic Ellipsometer (WVASE32, M-2000UTM, J.A. Co., Woollam, Lincoln, NE, USA). 250 and 900 nm range in visible region was used to analyze the samples at three different angles (65.70 and 75°). WVASE32 software was used to fit the data while using a Cauchy model. Homogeneity of solid electrolytes was evaluated with a HITACHI S-4700 field emission scanning electron microscope (FESEM). FTIR spectra were collected while using a spectrometer Spectrum 100 of Perkin Elmer with an ATR accessory (Attenuated Total Reflectance) with a resolution of 4 cm^−^^1^ in the range 4000–650 cm^−^^1^. The Raman spectra of the hybrids were recorded while using a Confocal Raman Microscope of WITec GmbH, Ulm, Germany (model alpha300), with a laser of 532 nm in the range 1600–200 cm^−^^1^.

Electrochemical AC Impedance Spectroscopy (EIS) using a BioLogic VMP3 Versatile Multichannel potentiostat/galvanostat with a two-electrodes device was used to measure the Li-ion conductivity. The experimental conditions were: 50 mV of amplitude voltage, 10^6^ and 0.1 Hz frequency range and acquisition data of 60 points. Membranes were sandwiched between two blocking electrodes of stainless steel in a glove box. The electrochemical active area of 0.01 cm^2^ was obtained while using silver conductive paint (Electrolube^®^, UK). Previous measurement, solid electrolytes were left at 100 °C for 24 h. 20 to 100 °C interval range was analyzed, keeping the system at least 1 h for each temperature in order to confirm that final temperature was achieved. The measurements were analyzed three times for each temperature to get reproducibility and the presented conductivity results are an average of those measurements. Li-ion conductivity *σ* of the samples was obtained Equation (1):

(1) $$\sigma \;=\;\frac{t}{R_{ct}\;S}$$

where *R_ct_* is the electrolyte resistance obtained from the intersection of the semicircle with the real impedance axis (fitted using a software program) and *t* and *S* are the thickness and electrode area of the sample, respectively. The conductivity data were plotted while using Arrhenius equation:

(2) σ = σ0.e(  − EakT)

where σ_0_ and k are the pre-exponential factor and the Boltzmann constant, respectively, *Ea* is the activation energy and σ is the ionic conductivity. The conductivity at room temperature for each solid electrolyte is obtained by interpolation in the Arrhenius equation, also obtaining the activation energy for the conduction process (*E_a_*).

The electrochemical stability windows (ESW) of the Solid Hybrid Electrolytes (SHE) was determined by cyclic voltammetry (CV) while using stainless steel (SS) as a working electrode and lithium metal as the counter and reference electrodes with the asymmetrical structure Li|SHE|SS while using a Swagelok^®^ type cell at scan rate of 5 mV s^−1^ from 0.5 to 7.0 V vs. Li/Li^+^.

The lithium-ion transference number (*t_Li_^+^*) of the solid hybrid electrolytes (SHE) at 25 °C was measured by a combination measurement of *ac* impedance and *dc* polarization while using a symmetric cell of Li metal|SHE|Li metal, as described by Evans [30], while using a Swagelok^®^ type cell. The surface of lithium metal was shaved with a scalpel before use. The cell was assembled in a glove box (H_2_O and O_2_ < 5 ppm). A *dc* voltage (300 mV) was firstly applied until a steady current was obtained (usually 14–16 h in this study), and the initial (*I*_ss_) and steady (*I*_o_) currents (μA), which flow through the cell, were measured. Simultaneously, the impedance spectra of the cell were recorded in the frequency range from 10^6^ to 0.01Hz, with an oscillation voltage of 10 mV, before and after the *dc* polarization, to obtain the initial (*R*_i_) and final (*R*_f_) resistances (Ω) of the electrolyte, and the initial (*R_1_^0^*) and final (*R_1_^ss^*) resistances (Ω) of the interfacial layers of the Li metal electrode/electrolyte. On the base of these values that were measured for the parameters above, the lithium-ion transference number (*t_Li_*^+^) was then calculated by Equation (2):

(3) $$t_{{Li}^{+\;}}\;=\;\frac{I_{ss}R_{f\;}\left[\Delta {V\;-\;I}_{0}R_{1}^{0}\right]}{I_{0}R_{i}\;\left[\Delta {V\;-\;I}_{ss}R_{1}^{ss}\right]}$$

## 3. Results and Discussion

### 3.1. Apareance of Solutions and Materials

Sols of GTT system are homogeneous, translucent, and have amber coloration; no precipitates or phase separation are observed. The pH of the sols is around 8 and the viscosity is between 4.3–4.8 mPa s as a function of TPTE concentration. Sols are stable in time, which conserves their rheological properties in periods exceeding one year.

**Figure 2:**
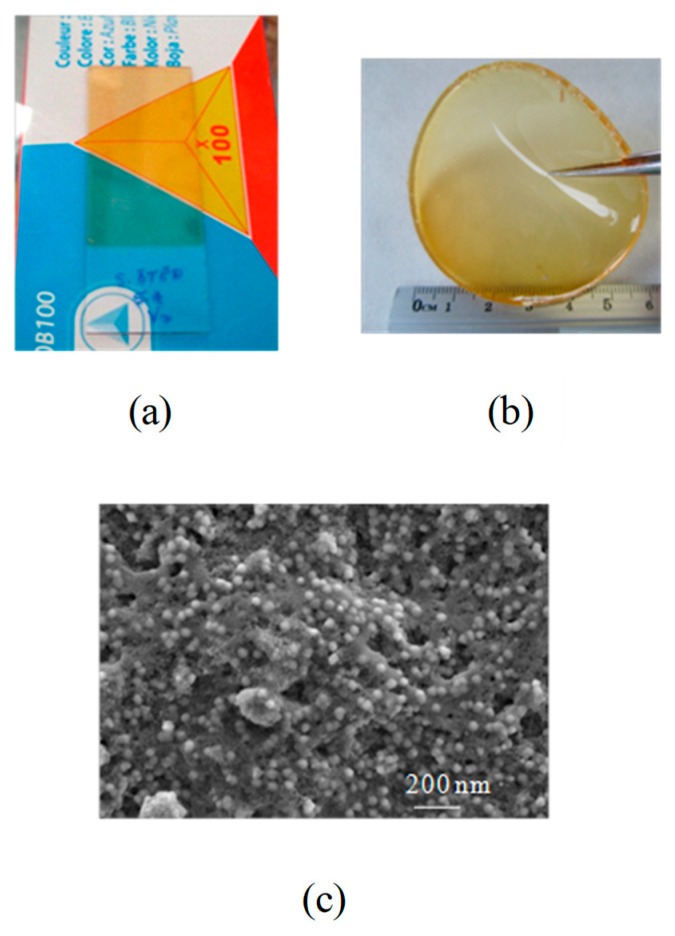
Representative photographs of hybrid, (**a**) Thin-film of GTT-2 composition and (**b**) self-supported membrane of GTT-2 composition, both treated at 100 °C in Ar atmosphere; and, (**c**) FE-SEM photographs of a fracture surface of GTT-3 hybrid electrolytes.

Homogeneous, slightly yellow, and transparent coatings that were deposited on glass substrates were obtained. Coating thicknesses oscillate between 0.1 and 1 μm for the different speeds of extraction while using the dip-coating process. Figure 2a shows an image of a coating of composition GTT-2 obtained by the dipping method and heat-treated at 100 °C for 12 h in an Ar atmosphere (inside the glove box). This temperature is enough to remove the solvents and water coming from sol-gel reactions, accelerates condensation reaction and to consolidate the hybrid structure without a loss of flexibility.

Self-supporting membranes that were obtained after the heat treatment at 100 °C have a yellow coloration, are homogeneous and transparent, without cracks, very flexible, and highly resistant to manipulation (Figure 2b). These adequate mechanical properties are due to the plastificant effect of LiTFSI salt, which also increases the amorphous domains of the material, and therefore prevents the crystallization of PEO chains [31]. Undoped sample (GTT-5) presents the same appearance but less flexibility when comparing with the doped samples. The thicknesses of the thermally treated and structurally consolidated membranes are around 400 μm as a function of the viscosity of the sols and the casting volume used. FE-SEM image of the fracture surface for sample GTT-3 (Figure 2c) shows a homogeneous material that formed by particles of 20–40 nm without phase separation, and low size porosity (5–10 nm), which is very important for allowing lithium mobility along the crosslinked hybrid network.

### 3.2. Structural Characterization

**Figure 3:**
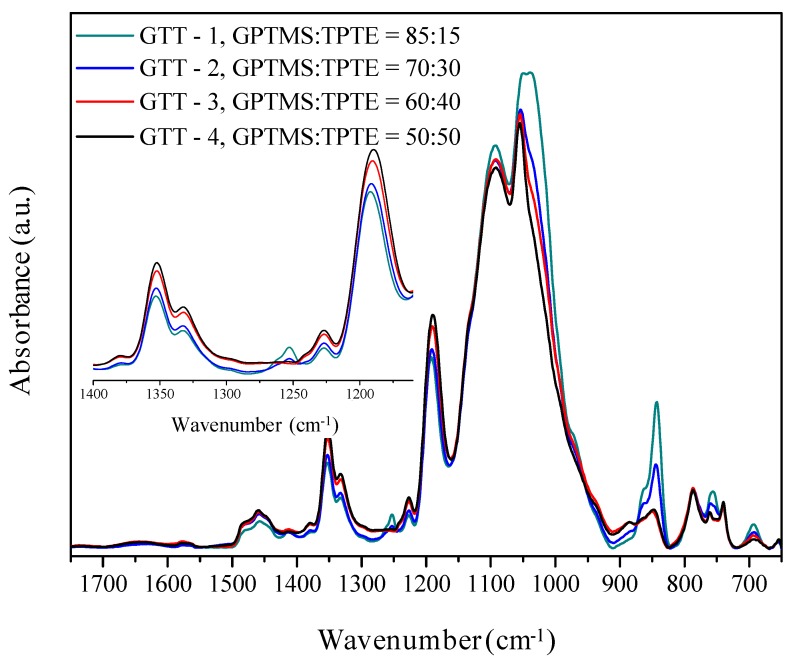
FTIR spectra of hybrid electrolytes heat-treated at 100 °C for 12 h.

Figure 3 shows the infrared spectra (FTIR) in ATR mode in the interval 1750–650 cm^−1^ of the GTT self-supporting membranes of different compositions with lithium, [Li +]/[O] = 0.10. Table 2 shows the main assignments for the GTT electrolytes and the precursors used in their synthesis [32,33,34,35,36,37,38,39,40].

Spectra confirm the formation of hybrid network and the adequate incorporation of the lithium salt in the structure of the hybrid. In the region 1400–1150 cm^−1^ (inset Figure 2), the doublet located at 1352/1332 cm^−1^ can be assigned to the anion TFSI^-^ vibration modes that correspond to the asymmetric mode ν_as_(SO_2_) [32,33,34]. Band at 1227 cm^−1^ attributed to ν_s_CF_3_ and the band at 1192 cm^−1^ associated with ν_a_CF_3_ that overlaps with the asymmetric stretching vibration band ν_as_(Si-O-Si) LO [32,33,34]. This band that is related to Li salt slightly increases its intensity with the organic precursor content, which could be due to the possibility that imide anions form complexes with the lithium that is also bound to the oxygen of the ethers [35]. On the other hand, the 868 and 846 cm^−1^ bands that are associated with cross-linking of the inorganic network (polycondensation) and the formation of SiO_4_ tetrahedra, respectively, increase with the content of inorganic precursor and is so more markedly in compositions with 70 and 85% of GPTMS [32,36,37]. This result is in accordance with highly crosslinking materials. In addition, peaks that are assigned to the lithium salt integrated in the hybrid are observed in the 780–720 cm^−1^ region related to the vibration modes ν_s_(S-N-S) [36,37,38]. The peak that is located at 740 cm^−1^ (ν_s_(S-N-S)) can be directly related to the formation of ion pairs, showing that the polyethylene oxide chains have a key role in the mechanisms of ionic mobility [35,39]. This band increases in intensity with the organic precursor content, which indicates the greater formation of ionic pairs in the electrolytes with higher TPTE content. This behavior is explained in part by the electrostatic interactions of the polyethylene oxide chains with the imide anions and the formation of complexes with Li^+^. It is also possible to see an increase in the intensity of the signal centered at 756 cm^−1^ in the sample with the highest inorganic precursor content (polycondensation). This band is assigned to the link voltage ν (S-N) and it is related to the formation of complexes or aggregates of the type (CF_3_SO_2_)_2_N-Li^+^—polymer [37,40].

The band around ~1200 cm^−1^ assigned to the vibration mode ν_as_(Si-O-Si)_LO_ increases in intensity with GPTMS content [34,39]. The signal centered at 1046 cm^−1^, which is attributed to the asymmetric modes of the Si-O-Si/C-O-C bonds, and another band at 1092 cm^−1^ related to antisymmetric stretching mode of Si-O-Si bond, are also more intense with higher content of GPTMS precursor, which suggests the formation of complexes between the lithium salt and the chains of aliphatic ether groups (-C-O-C-) resulting from the polymerization reactions of the epoxide rings of the GTT precursor [26,36,37,38]. Moreover, a shoulder is observed at ~1136 cm^−1^ (ν(-Si-O-Si-O-)/ν_s_(SO_2_)), which seems to indicate that LiTFSI salt affects to the hybrid structural order and the degree of cross-linking [37]. The band at 956 cm^−1^ that is attributed to the vibration of the Si-OH groups has decreased their intensity almost completely (shoulder), which demonstrates that the TMES precursor has effectively blocked such groups [39].

**Figure 4:**
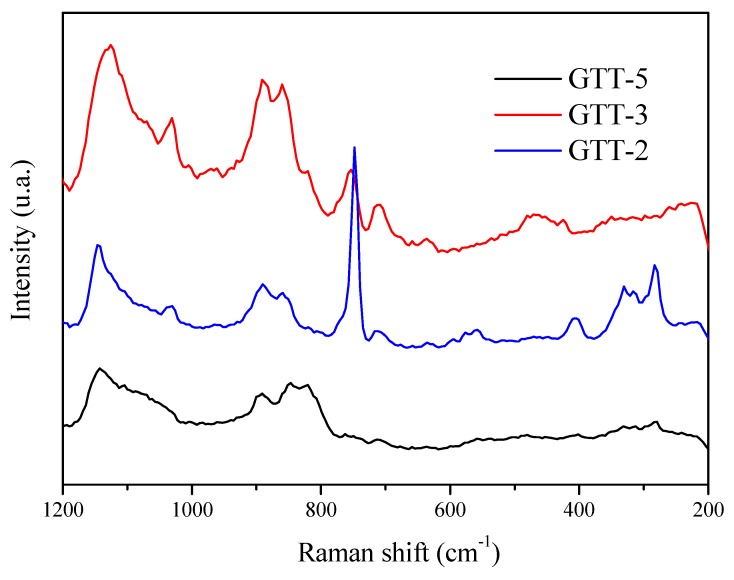
Raman spectra of hybrid electrolytes GTT-2, GTT-3, and GTT-5, heat-treated at 100 °C for 12 h.

Raman spectra were performed in order to complement the structural characterization of the hybrid solid electrolytes. Figure 4 shows the Raman spectra of hybrid solid electrolytes heat treated at 100 °C for 12 h in the range 1200–200 cm^−1^ for different GTT compositions.

The incorporation of Li in GTT-2 and GTT-3 compositions is proven with the presence of three peaks at 744, 748, and 752 cm^−1^, which correspond to symmetric vibration mode of the bond ν_a_CF_3_ and ν_s_(SNS^−^), related to free ion pairs Li^+^-TFSI^−^ [40,41]. The intensity of this peak increases with the content of organic precursor (EGDE), which seems to indicate those parts of the TFSI^-^ anions are mostly free. Another interesting region, 270–330 cm^−1^, is related to SO_2_ and CF_3_ wagging mode from LiFTSI salt [42]. Organic polymerization via epoxyde ring opening to form polyethylene oxide chains was confirmed by the presence of two peaks at 1144 and 1122 cm^−1^, which were assigned to stretching vibration ν (C-O-C) and v C-O modes, and a coupled vibration of ν (C-C) with δCH_2_, respectively. These bands overlap with vibration stretching band of ν_s_ SO_2_ causing a wide band.

The degree of cross-linking can be studied through the bands located at ~1136 cm^−1^ and ~840 cm^−1^, which is assigned to the vibrations ν (-Si-O-Si-) [41]. These bands shift to ~1144 (wide) and ~857 cm^−1^ in the sample GTT-2 with respect to GTT-3, because a greater proportion of TPTE generates a higher degree of cross-linking (polycondensation). The band that appears at 410 cm^−1^ is related to stretching vibration of SiO_2_ bonds, which indicates a high degree of polycondensation in the structure [43,44,45].

### 3.3. Electrochemical Characterization

**Figure 5:**
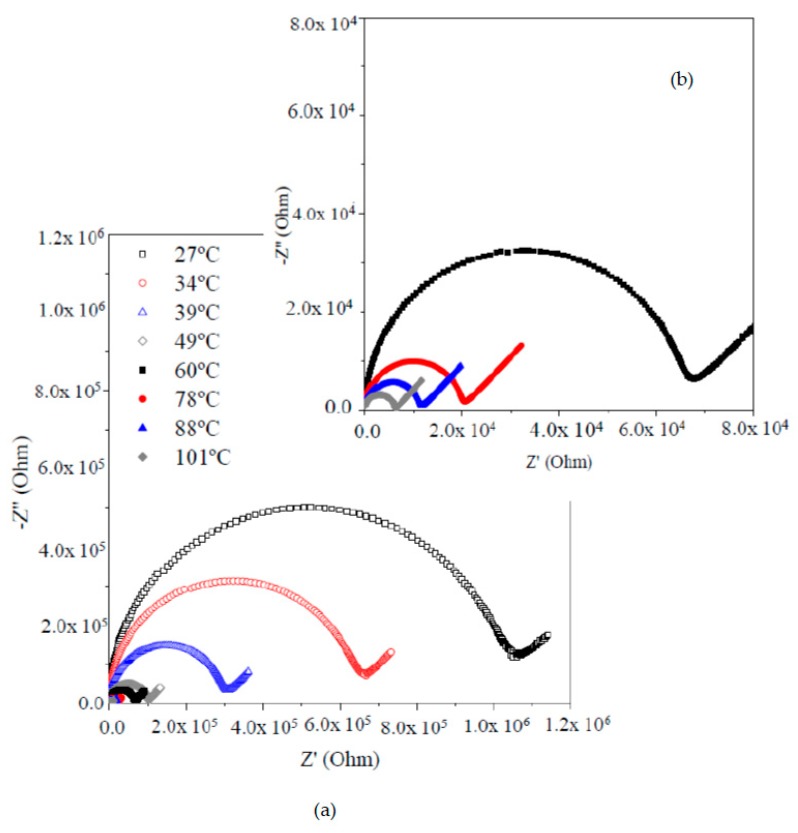
(**a**) Nyquist plots (two-probe method) for GTT-3 composition heat-treated at 100 °C for 12 h. (**b**) Amplification of the Nyquist spectra at high frequency area.

Figure 5 shows the Nyquist impedance plot at different temperatures of a self-supported membrane of GTT-3 composition, while using a two-probes method.

For all of the temperature range, a similar response is observed, a semicircle that crosses out the origin at high frequencies, related to the electrical response of the material, followed by a region at low frequencies, in which a line appears with an angle of approximately 45°, which either represents a Warburg impedance associated with the diffusion processes or the beginning of a second time constant that is associated with the processes that take place at the membrane-electrode interfaces. Resistance was obtained by the interpolation of the semicircle with the abscise axis. The amplitude of the semicircle decreases (increasing Li-ion conductivity) when the temperature increases (Figure 5b), which indicates that the electrochemical process following an Arrhenius behavior (activated process).

**Figure 6:**
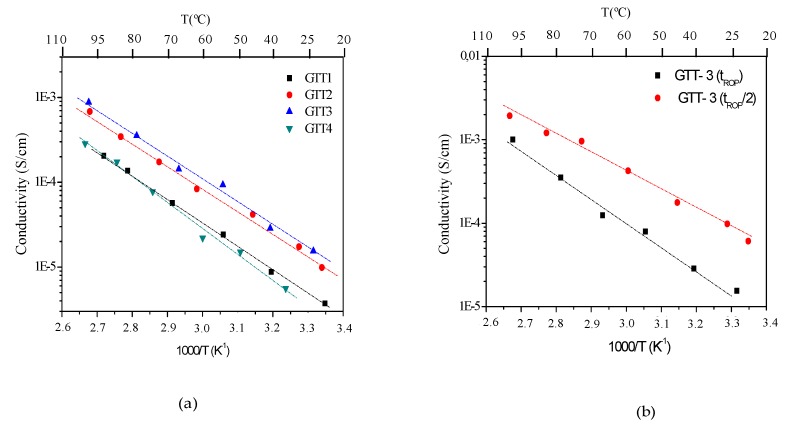
Conductivity measurements of GTT with [Li^+^]/[O] = 0.10 (**a**) as a function of Trimethylolpropane triglycidyl ether (TPTE) organic precursor and (**b**) with a different organic polymerization time.

A study of the conductivity as a function of temperature for the GTT system has been carried out, varying the GPTMS: TPTE ratio and keeping the Li content constant ([Li^+^]/[O] = 0.10) (Figure 6a). Table 3 summarizes the conductivity and activation energy of the GTT solid hybrid electrolytes.

As can be observed, the conductivity values at room temperature are between 10^−5^ and 10^−4^ S cm^−1^ and the activation energy is around 0.6 eV. From Figure 6b, it can be deduced that the conductivity values follow the same trend; conductivity increases with TPTE content up to 0.67 M (GTT-3) and then decreases slightly. This seems to indicate that the increase in epoxide groups in GTT-4 generates a high crosslinking hybrid structure with less availability of ether groups (steric effect) affecting the segmental motion of PEO chains and ion pairs Li*^δ^*^+^ --- O*^δ^*^-^–R [46]. The values of conductivity are quite similar that the reported previously for the GTE hybrid electrolyte system [28], in which a diepoxi monomer was used (GTED) instead of TPTE precursor.

The GTT hybrid materials were synthesized following the synthesis strategy that is based on Popall et al. [24] and for polymerization times epoxide ring opening were calculated based on the contents of TPTE hybrid precursor (10–14 h). However, the degree of crosslinking of the hybrid network has been greater than expected, which reduces the ionic conductivity of the material. Therefore, a reduction (half) of the organic polymerization time was tested in the GTT-3 composition, with similar epoxide group content, n = 3 (Figure 6b). It is observed that the conductivity exhibits higher values for shorter polymerization times (T_ROP_/2) than those that were proposed by Poppal, reaching conductivity values of 9.5 × 10^−3^ and 2.2 × 10^−3^ S cm^−1^ at room temperature and 60 °C, respectively. Therefore, it is demonstrated that a lower crosslinking degree significantly influences solid electrolytes ionic conductivity; more open structures [47], with free volume for a greater dispersion of Li^+^ [36] and good availability of ether groups [36,48] exhibit higher conductivities. However, it is necessary to achieve a compromise between hybrid structure and ionic conductivity and the availability of Li ions in the structure [49]. The use of GTT precursor (tri-epoxi) with an estrict control of polymerization grades led to a crosslinking electrolyte with better ionic conductivity (more than two order of magnitude) than the reported for GTE electrolytes (GTED, diepoxi) precursor [28].

**Figure 7:**
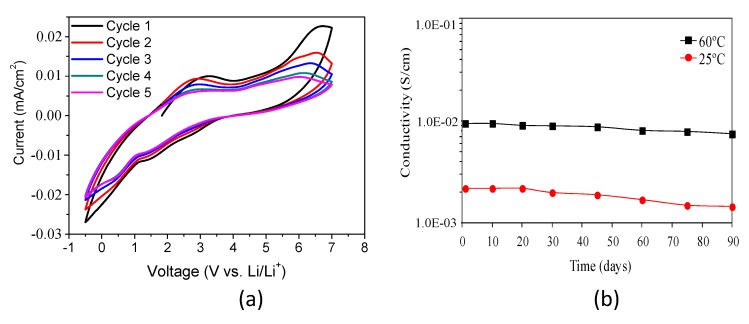
(**a**)Electrochemical stability window for GTT-3 composition using a SS/solid electrolyte/Li cell at room temperature with dE/dt = 5 mV s^−1^. (**b**) Ionic conductivity of GTT-3 (T_ROP_/2) composition versus time at different temperatures (25 and 60 °C).

The electrochemical stability window of these materials has been analyzed in order to know the electrochemical stability when they are in contact with the electrode. Figure 7a shows the first five cycles of cyclic voltammetry of the composition GTT-3; a scan rate of 5 mV s^−1^ was used in the range −0.5 and 7.0 V vs. Li/Li^+^. Anodic and cathodic peaks appear at the extremes of the voltage range, keeping good symmetry between them, which means that the process is highly reversible. The electrolyte is stable up to a potential of around 5 V, because a considerable flow of current is detected from this point, related to the beginning of the electrolyte decomposition processes [25]. A broad oxidation peak is also observed in the anodic branch around 2.2–3.2 V, which may be due to the oxidation of surface impurities in the hybrid [50]. However, this peak has a very small relative intensity if one takes into account the scale of measurement (≤0.005 mA/cm^2^), being negligible. The GTT hybrid solid electrolyte system presents a wide electrochemical stability window in the range 1.5–5 V versus Li/Li^+^, which is similar to other polymer electrolytes [51,52,53,54,55,56,57].

The long-term stability of the solid hybrid electrolytes was investigated by monitoring the time dependence of the ionic conductivity at different temperatures: 25 and 60 °C. The compatibility between solid electrolyte and lithium anode, which can be evaluated by the interfacial impedance (Ri), is a key factor for application in a lithium ion battery. Figure 7b displays the variation of ionic conductivity as a function of time for the solid hybrid electrolyte GTT-3 (T_ROP_/2) composition. It is clear that the ionic conductivity of the hybrid solid electrolyte was not practically affected by the time of used obtaining 7.5 × 10^−3^ and 1.5 × 10^−3^ S/cm after 80 days for 25 °C and 60 °C, respectively. This slight decrease it is probably due to the formation of the passivation layer (SEI). Therefore, one might conclude that these solid hybrid electrolytes show moderate stability against Li in agreement with the capacity fading during cycling.

The ion transfer number gives an idea of the phenomena related to the association of the ions in the material and it determines the formation of the passivation layer (SEI) [58]. The modified method of Evans et al. [30] was used to determine the ion transfer numbers (Table 3). The typical transport number values at room temperature for polymeric solid electrolytes are usually around 0.5 [25,46,59,60]. The results for the GTT system are similar, reaching a maximum of 0.53 for GTT-3 (T_ROP_/2) composition, in accordance with the ionic conductivity. This indicates that the ion conduction mechanism in this case is quite similar to polymer electrolytes. This parameter is closely related to the availability of ion pairs in the electrolyte structure, as verified by FTIR and Raman. Composition GTT-3 (T_ROP_/2) has a suitable cross-linking, which is sufficient for obtaining a cross-linked hybrid structure, but with mobility of ether chains and availability of the lithium salt to form ion pairs.

**Figure 8:**
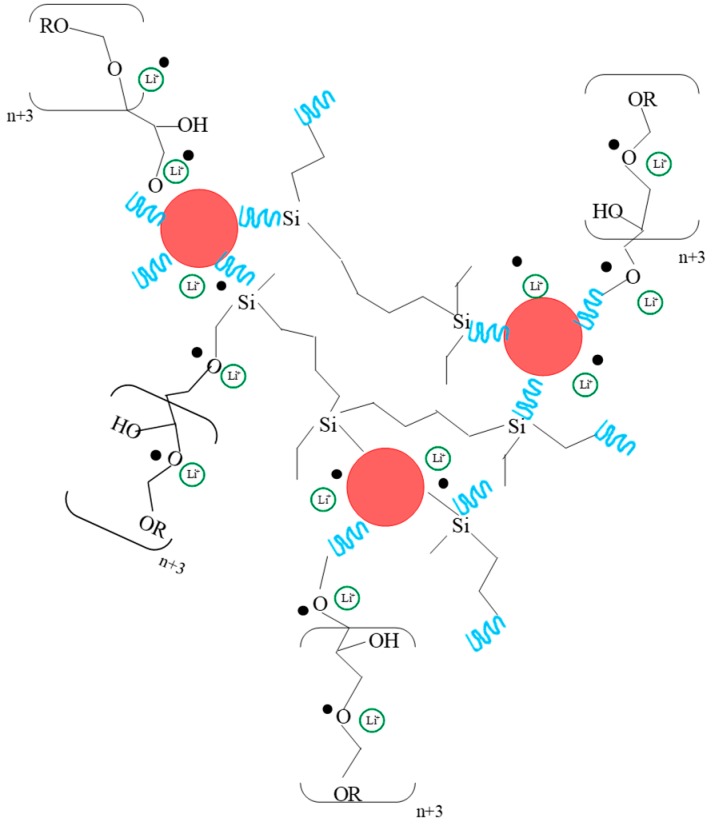
Schematic representation of hybrid material obtained using TPTE triepoxy precursor (in red inorganic domains).

Figure 8 shows a schematic representation of hybrid organic inorganic structure that is based on the results previously discussed. The organic co-polymerization generates a network of oligomers, in which, in a second stage, the inorganic environments that are included in this network are incorporated. The precursor GTT, with three epoxide groups, generates a highly crosslinked structure that can limit the segmental movement of the organic chains, damaging the processes of ionic mobility and, therefore, the conductivity. For short polymerization times, an open hybrid structure that is facilitated by the length of the precursor chain is obtained. This hybrid structure presents greater availability for the ether groups of the polymer chains and a greater degree of movement, which facilitates ionic movement. Most Li^+^ are free (FTIR and Raman), interacting with the ether oxygen’s of the structure.

## 4. Conclusions

Nano-structured hybrid organic-inorganic electrolytes have been prepared with covalent bond between their components by the sol-gel technique. Li ions are mostly free interacting with the ether oxygens of the structure (FTIR and Raman). The GPTMS-TMES-TPTE (GTT) system synthesis strategy was optimized in terms of epoxi ring opening polymerization time. It has been found that the content of organic precursor directly influences the ionic conductivity of the material. For composition GTT-2 and GTT-1 (with contents greater than 60 mol% of TPTE (trimethylolpropane triglycidyl ether), the ionic conductivity is decreased due to highly cross-linked structures that limit the segmental movement of the organic chains. The prepared solid organic-inorganic hybrid electrolytes have suitable values of ionic conductivity of 10^−4^ S/cm at room temperature for the composition GTT-3. The conductivity increases by up to two orders of magnitude for compositions with half of the epoxy ring opening polymerization time, GTT-3 (T_ROP_/2). This composition has a transport number that is close to 0.53 and the electrochemical window had a wide stability range between 1.5–5 V (*vs.* Li/Li^+^), which allows for its implementation in batteries with high energy density.

## Figures and Tables

**Table 1:** Compositions of organic-inorganic hybrid electrolytes in GTT systems.

Composition	Composition (mol)						
	GPTMS	TPTE	n-MI	EtOH	LiTFSI	[Li^+^]/[O]	GPTMS:TPTE %mol
GTT-1	1.0	0.18	0.06	11.8	0.15	0.10	85:15
GTT-2	1.0	0.43	0.09	14.3	0.23	0.10	70:30
GTT-3	1.0	0.67	0.12	16.7	0.30	0.10	60:40
GTT-4	1.0	1.00	0.16	20.0	0.40	0.10	50:50
GTT-5	1.0	0.43	0.09	14.3	0.00	0.00	70:30

**Table 2:** FTIR assignments of the main absorption bands for sol-gel precursors and GTT hybrid network [32,34,35,37,38,39,40].

GPTMS	TPTE	Hybrid Network	Assignments	Reference
		1643	ν(C = C) (CH = CH of *n*-MI)	[37]
1480–1370		1480–1370	δCH_3_ of GPTMS	[34,37]
	1462–1456	1454	δ_as_HCH/CH_2_ *scissor*/δ(CH_2_)	[34]
1420		1410–1414	δCH_2_/ν_as_(C-O-O)	[39]
		1352	CH_2_ *wagg*/ν_as_(SO_2_) of LiTFSI	[38]
		1332	ν_as_(SO_2_) of LiTFSI	[35]
		1273–1294	ν (C-Si-O)	[26,34]
1254	1253	1253	δ(Si-CH_2_)	[34]
1260–1240	1250; 909–907		ν epóxido	[26]
		1227	ν_s_CF_3_ of LITFSI	[35,37]
		1194–1189	ν_as_(Si-O-Si)_LO_	[26,37]
		1136	ν(-Si-O-Si-O-) (crosslinking)/ν_s_(SO_2_) of LiTFSI	[32,34,37]
	1093	1092	ν_as_(Si-O)/ν_as_(C-O)/ν_as_CF_3_ of LiTFSI	[32,34,35]
		1046	ν_s_(C-O-C)/ν_as_(Si-O-Si)	[32,34]
	993–989	956	ν(C-O-C)	[34,39]
		884–880	δCH_2_/ν(C-OH)	[35,37]
		854–852	δ(Si-O)	[37]
		846	ν(-Si-O-Si-) (crosslinking)	[37]
816			ν(Si-O-CH_3_)	[26,36]
		795–787	ν_s_(Si-O-Si)	[36]
		756*	ν(S-N) of LiTFSI	[35,37]
750			δ(Si-O-CH_3_)	[39]
		740	ν_s_(S-N-S) of LiTFSI	[35,37]

**Table 3:** Ionic conductivity and activation energy of GTT hybrid solid electrolytes.

Composition	*σ*_25 °C_ (S/cm)	*σ*_60 °C_ (S/cm)	*Ea* (eV)	*t_Li_*^+^ (25 °C)
GTT-1	4.9 × 10^−6^	4.3 × 10^−5^	0.62	0.36
GTT-2	1.3 × 10^−5^	1.0 × 10^−4^	0.56	0.40
GTT-3	1.8 × 10^−5^	1.4 × 10^−4^	0.51	0.45
GTT-4	3.1 × 10^−6^	3.8 × 10^−5^	0.67	0.31
GTT-5	~10^−11^	~10^−11^	-	-
GTT-3_(TROP/2)_	1.3 × 10^−4^	1.4 × 10^−3^	0.49	0.53
